# Urinary Metabolites during Pregnancy: A Literature Review

**DOI:** 10.1021/acsnutrsci.5c00028

**Published:** 2026-02-23

**Authors:** Shiyi Y. L. Xie, Amar Hamdan, Sarah S. Comstock

**Affiliations:** Department of Food Science and Human Nutrition, 3078 Michigan State University, East Lansing, Michigan 48824, United States

**Keywords:** urinary metabolites, prepregnancy BMI, pregnancy, metabolomics, trimester

## Abstract

Urinary metabolites provide information regarding metabolic changes and environmental exposure throughout pregnancy. A narrative literature review was conducted by using PubMed and Web of Science. Included studies enrolled pregnant women aged ≥18 years and analyzed urinary metabolites in relation to prepregnancy body mass index (BMI) or trimester of pregnancy. Environmental and dietary exposures were also considered. Thirteen studies met the criteria. Women with a higher prepregnancy BMI have unique urinary metabolite profiles, including lower levels of glucogenic amino acids and long-chain acylcarnitines, reflecting disturbances in amino acid and lipid metabolism. The third trimester was marked by elevated urinary cortisol, carboxylic acids, glycerolipids, and steroid derivatives. Environmental exposures such as diet, phthalates, metals, and parabens were linked to distinct urinary metabolite patterns. More comprehensive analyses of urinary metabolites during pregnancy in diverse populations are needed to identify early metabolic risks and develop targeted prenatal interventions to improve maternal and fetal health outcomes.

## Graphical abstract

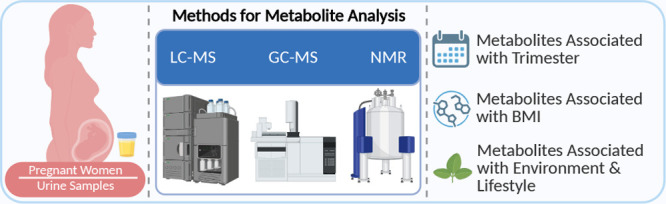

## Introduction

In recent years, the prevalence of obesity among women of childbearing age has continued to rise globally.^1^ In the United States, prepregnancy obesity has risen from 26.1% to 29.0% between 2016 and 2019, with the most significant upward trend in a predominantly young female population.^2^ The trend has continued in more recent years, rising to 32% in 2023.^3^ The rise has attracted widespread attention in the public health field, as prepregnancy obesity has been recognized as an important factor affecting maternal and fetal health outcomes.^4^

A high prepregnancy body mass index (BMI) is strongly associated with a range of adverse perinatal outcomes, including gestational diabetes, preeclampsia, hypertensive disorders, and increased cesarean delivery rates.^5^ For example, women with obesity (≥30.0 kg/m^2^) have a significantly higher risk of developing preeclampsia compared to women with a BMI in the normal range (18.5–24.9 kg/m^2^), while women who are underweight (<18.5 kg/m^2^) have a relatively lower risk.^6,7^ Furthermore, women with prepregnancy obesity had about 2.7 times the risk of developing gestational diabetes mellitus (GDM) than women with a normal BMI.^8^ Additional clinical solutions are needed to prevent these conditions and to improve outcomes for those experiencing such conditions during pregnancy.

Metabolites are a direct reflection of the body’s metabolic state and have emerged in recent years as an important tool for identifying the risk of disease in pregnancy.^9^ Particularly in studies of common metabolic diseases such as GDM, specific metabolites in urine have been found to be significantly altered before the onset of clinical symptoms.^10,11^ Identifying such biomarkers through early screening and intervention could lead to improved health outcomes, especially for women with higher prepregnancy BMI.^12^

During pregnancy, the body undergoes a series of metabolic adaptations to support fetal growth and development,^13^ including changes in glucose metabolism, lipid metabolism, and insulin sensitivity.^14–16^ However, prepregnancy obesity may interfere with these physiologic adaptive processes, leading to metabolic disturbances such as increased insulin resistance, elevated inflammatory factors, and lipid abnormalities.^14,17^ These abnormalities not only increase the risk of pregnancy complications but may also have long-term adverse effects on fetal development.^15^ Therefore, exploring the effects of prepregnancy obesity on metabolic processes is a key basis for understanding its association with pregnancy outcomes.

The stage of pregnancy is also an important factor influencing the metabolic status of the mother.^18^ As pregnancy progresses, the body faces significant changes in metabolic demands and hormone levels during each trimester, affecting the metabolic pathways and metabolite composition in the body.^18–20^ Many of these changes are detectable by measuring metabolites in urine, which serves as a noninvasive medium reflecting both endogenous metabolic activity and physiological adaptations.^21^ Therefore, studying urinary metabolites across different pregnancy stages is critical for capturing trimester-specific metabolic signatures.

In addition, environmental exposures may also modulate metabolism during pregnancy.^22^ Dietary patterns, metal exposure and chemicals such as phthalates alter maternal metabolite profiles by influencing metabolic pathways and, in turn, metabolites.^22,23^ These changes are often reflected in urinary metabolite profiles, which respond dynamically to both internal processes and external exposures.^21^ Thus, urinary metabolomics offers a practical approach for assessing how environmental factors shape maternal metabolic health during pregnancy.

In this review, prepregnancy BMI was the primary exposure and pregnancy trimester was the secondary exposure of interest, while environmental exposures were also examined (Table 1). Analysis of urinary metabolites associated with these factors using a variety of detection methods is crucial to understanding metabolomic signatures during pregnancy. Urine is readily available, and the urinary metabolome has been shown to be relatively stable across common storage conditions and additives, enhancing its reliability for biomarker discovery.^24^ This review provides an overview of existing studies analyzing the urinary metabolome during pregnancy and emphasizes the importance of metabolic profiles in the prenatal period.

**Table 1:** Description of the Population, Exposures, Comparators, and Outcomes Considered in This Review^a^

Population	• Over 18 years old
	• Pregnant
	• From anywhere in the world
	• Can have health conditions (e.g., gestational diabetes)
Exposures	• Prepregnancy BMI (primary exposure factor)
	• Pregnancy trimester
	• Methods for detecting urinary metabolites
	• Environmental: chemicals, metals, dietary intake
Comparators	• Comparisons of metabolite changes across prepregnancy BMI groups (normal, overweight, obese, with obese as the comparator)
	• Comparisons across trimesters (with the third trimester as the comparator)
	• All methods (e.g., GC-MS, NMR) are compared to the LC-MS method
	• Comparisons are made between individuals exposed to chemicals, metals, and dietary intake and those not exposed
Outcome	• Urinary metabolite levels

a Abbreviations: BMI, body mass index; GC-MS, gas chromatography–mass spectrometry; NMR, nuclear magnetic resonance; LC-MS, liquid chromatography–mass spectrometry.

## Method

### Databases and Search Strategy

PubMed^25^ and Web of Science^26^ were searched, as both are widely used in the fields of biomedical and health research and provide access to numerous high-quality, peer-reviewed studies. A search strategy using Boolean operator combinations (e.g., “urine metabolites AND pregnancy AND BMI NOT reviews” and “metab* AND BMI AND Urin* AND pregnancy NOT review”) as well as additional filters such as age (adults 18+) and no limit to publication date was applied. These filters were applied to optimize the retrieval of studies that cover all aspects of the research questions. Given the relatively small number of eligible studies and the heterogeneity in study designs, exposures, and outcomes, we used a structured narrative review approach rather than a PRISMA-guided systematic review while still applying predefined search terms and inclusion criteria. The full search strategy can be found in Table 2.

**Table 2:** Search Strategies

	PubMed	Web of Science
search date	from 07/2024 to 10/2024	from 07/2024 to 10/2024
date range	no limit	no limit
search terms	(“urine metabolites” AND pregnancy AND BMI NOT reviews); (metab* AND BMI AND Urin* AND pregnancy NOT review); (untargeted AND metab* AND Urin* AND pregnancy NOT review); (pregnan* AND urin* AND metab* AND BMI)	(metab* AND BMI AND Urin* AND pregnancy NOT review)
filters	adults (18+)	adults (18+)

### Inclusion and Exclusion Criteria

The inclusion criteria for this review were focused on selecting studies published without a date restriction. Studies are drawn from a global context. All selected studies were published in English, but no restrictions were placed on the language spoken by the study participants. The target population for this review includes pregnant individuals aged 18 years and older with only primary research studies included. Studies were excluded from this review if they lacked primary data (such as meta-analyses, reviews, commentaries) or if they did not focus on pregnant individuals aged 18 years and older. Additionally, studies were excluded if they did not provide data on urinary metabolites or their associations with prepregnancy BMI, pregnancy trimester, or environmental exposures. Additional exclusion criteria included poorly defined methodology such as unclear detection methods.

### Study Selection Process and Data Extraction

A two-stage study selection process was used to ensure the relevance and quality of the literature included (Figure 1). In the initial screening phase, titles and abstracts of identified studies were reviewed to assess their relevance to the main variables of prepregnancy BMI, pregnancy trimester, and environmental exposures. Studies that did not mention these variables in the title or abstract were excluded. For the secondary screening phase, the methods and results sections of each study were examined in detail to verify the study design and ensure that it met the inclusion criteria. Only studies that directly addressed relevant urinary metabolites, based on the main variables, were kept for the final analysis. Data were then extracted by collecting basic information to contextualize and compare research results.

**Figure 1:**
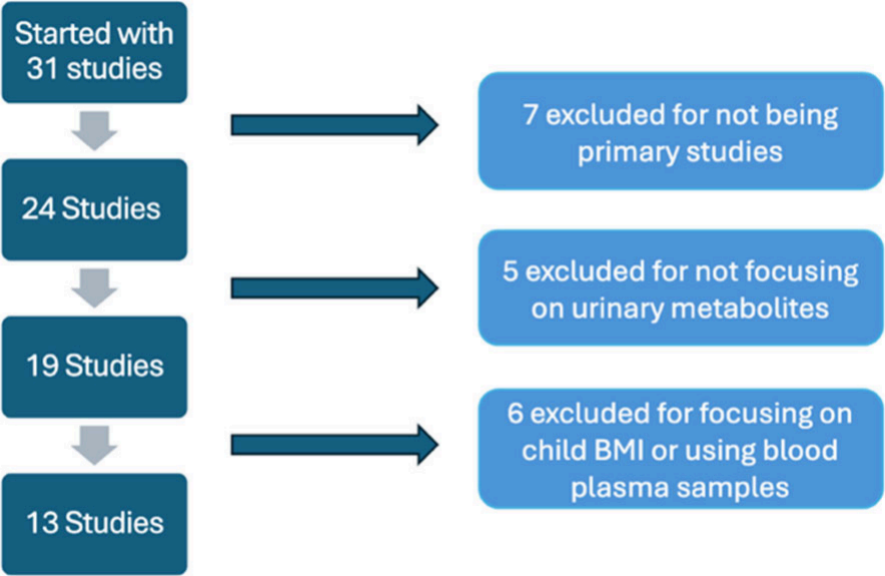
Flowchart of studies included in the review.

### Study Variables

The primary study variables were selected to investigate their relationships with the dependent variables of urinary metabolites. Prepregnancy BMI was the primary exposure and pregnancy trimester was the secondary exposure. These were chosen due to their well-established roles in influencing metabolic adaptation during pregnancy. Environmental exposures, including dietary intake, metals, and chemicals, such as phthalates, were also examined as exposures since they were expected to impact urinary metabolite profiles. Finally, detection methods for urinary metabolites were also considered, with the objective of comparing the types of metabolites identified by different methods such as liquid chromatography–mass spectrometry (LC-MS), gas chromatography–mass spectrometry (GC-MS), and nuclear magnetic resonance (NMR). Comparing the dependent variables, urine metabolites, by the independent variables (BMI, trimester, exposures) can provide an understanding of the factors influencing the urinary metabolite profiles in the prenatal period.

### Search Results

The literature search resulted in a total of 13 primary research studies that met the inclusion criteria (Figure 1). These studies analyzed various aspects of urinary metabolite profiles in pregnant women. Among the included studies, three papers focused on the association between prepregnancy BMI and urinary metabolites, four papers examined metabolites during different pregnancy trimesters, six different analytical techniques, including LC-MS, GC-MS, and NMR, were used to detect urinary metabolites, and six studies explored the effects of environmental exposures such as dietary patterns, metal exposure, and chemical exposure on urinary metabolites (Table 3). Sample sizes of the studies included in this review vary greatly, ranging from as few as 27 participants to as many as 1221 participants. Data from a total of 4491 participants is included in this review. Participants resided in the United States of America (USA, *n* = 6 studies), China (*n* = 3 studies), Mexico (*n* = 1 study), Canada (*n* = 1 study), Australia (*n* = 1 study), and Spain (*n* = 1 study).

**Table 3:** Overview of the 13 Papers Included in This Review

title	year published	metabolomic analysis method	sample size	study design	belong to which part of results	study location
Longitudinal Associations of Pre-Pregnancy BMI and Gestational Weight Gain with Maternal Urinary Metabolites: An NYU CHES Study^27^	2022	HPLC/FIA-MSMS	652	longitudinal cohort	BMI, trimester, detection method	New York, USA
Urinary Cortisol Is Lower in Pregnant Women with Higher Pre-Pregnancy BMI^28^	2023	competitive enzyme immunoassay	32	longitudinal observational study	BMI	Pennsylvania, USA
BMI-Specific Inflammatory Response to Phthalate Exposure in Early Pregnancy: Findings from the TMCHESC Study^29^	2023	GC-MS	394	cohort study	BMI, detection method	Tianjin, China
A Longitudinal Study of Plasma and Urinary Cortisol in Pregnancy and Postpartum^20^	2011	liquid chromatography–tandem mass spectrometry	47	longitudinal study	trimester	Melbourne, Australia
Urinary Metabolites Altered during the Third Trimester in Pregnancies Complicated by Gestational Diabetes Mellitus: Relationship with Potential Upcoming Metabolic Disorders^30^	2019	ultraperformance liquid chromatography–mass spectrometry	35	cross-sectional study	trimester	San Luis Potosi, Mexico
First and Second Trimester Urinary Metabolic Profiles and Fetal Growth Restriction: An Exploratory Nested Case-Control Study within the Infant Development and Environment Study^31^	2018	NMR spectroscopy	158	case-control study	trimester	Minneapolis, MN; Rochester, NY; San Francisco, CA; and Seattle, WA, USA
Maternal Pre-Pregnancy BMI Influences the Associations between Bisphenol and Phthalate Exposures and Maternal Weight Changes and Fat Accumulation^32^	2024	HPLC–high resolution mass spectrometry	318	prospective cohort study	detection method	Canada
Associations between the Maternal Exposome and Metabolome during Pregnancy^22^	2023	ultrahigh-performance liquid chromatography–high-resolution accurate mass spectrometry (UHPLC–HRMS)	1024	cohort study	detection method	Jiangsu Province, China
Effects of a Mediterranean Diet Intervention on Maternal Stress, Well-Being, and Sleep Quality throughout Gestation-The IMPACT-BCN Trial^33^	2023	liquid chromatography-tandem mass spectrometry (LC-MS/MS)	1221	randomized clinical trial	exposures	Barcelona, Spain
Associations between the Gut Microbiota, Urinary Metabolites, and Diet in Women during the Third Trimester of Pregnancy^34^	2022	LC-MS	27	cross-sectional study	exposures	Michigan, USA
Urinary Paraben Concentrations and Associations with the Periconceptional Urinary Metabolome: Untargeted and Targeted Metabolomics Analyses of Participants from the Early Pregnancy Study^35^	2023	ultrahigh-performance liquid chromatography–quadrupole time-of-flight mass spectrometry	42	case-control study within a prospective cohort study	exposures	North Carolina, USA
Prenatal Exposure to Mixtures of Phthalates, Parabens, and Other Phenols and Obesity in Five-Year-Olds in the CHAMACOS Cohort^36^	2021	isotope-dilution high-performance liquid chromatography–electrospray ionization tandem mass spectrometry	309	longitudinal cohort study	exposures	California, USA
Urinary Metabolomics Reveals Novel Interactions between Metal Exposure and Amino Acid Metabolic Stress during Pregnancy^37^	2018	inductively coupled plasma mass spectrometry	232	prospective cohort study	exposures	Wuhan, China

## Results

### Detection Methods

The typical methods used for metabolite analysis are liquid chromatography–mass spectrometry (LC-MS), gas chromatography–mass spectrometry (GC-MS), and nuclear magnetic resonance spectroscopy (NMR). Different detection methods can influence which metabolites are identified, so it is crucial to understand their differences.^38^ Studying the distinctions between methods can help researchers better understand metabolic data, which can improve the development of healthcare plans and perinatal outcomes.

Different analytical methods (Table 4) vary in their ability to detect specific urinary metabolites, with LC-MS often serving as the reference method due to its broad detection capabilities and high sensitivity. High-performance liquid chromatography–mass spectrometry (HPLC-MS) and flow injection analysis–mass spectrometry (FIA-MS) detect and quantify a wide range of metabolites—including amino acids, acylcarnitines, phosphatidylcholines, and sphingolipids—making them a comprehensive tool for targeted metabolomic studies.^27^ Additionally, LC-MS can also be used to measure urinary cortisol levels.^20^ In contrast, GC-MS is commonly used for detecting small, volatile compounds such as phthalate metabolites, including mono­(2-ethylhexyl) phthalate (MEHP), mono­(*n*-butyl) phthalate (MBP), and monoethyl phthalate (MEP), due to its superior capability for quantifying low molecular weight compounds.^29^ Although, HPLC-MS is capable of detecting smaller metabolites, such as phthalates and bisphenols.^32^ NMR spectroscopy, while less sensitive than mass spectrometry methods, excels in providing detailed structural information and can detect a variety of metabolites, such as carboxylic acids and certain exploratory metabolites like 1-Methylnicotinamide and sphingolipids.^31^ The ultrahigh-performance liquid chromatography Ultimate 3000 system-Q Exactive hybrid quadrupole–orbitrap high-resolution mass spectrometry (UHPLC-QE-HRMS) further expands the analytical range, allowing for the detection of additional environmental chemicals and complex exposome analysis, including pesticides, phthalates, and antimicrobial agents.^22^ In conclusion, each analytical method has distinct strengths and limitations in detecting urinary metabolites, making it suitable for different research objectives. LC-MS stands out for its broad detection range and high sensitivity, while GC-MS is ideal for small, volatile compounds. NMR provides structural insights despite lower sensitivity, and UHPLC-QE-HRMS offers expanded capabilities for the detection of complex environmental chemicals.

**Table 4:** Comparison of Urinary Metabolite Detection Methods^21,39^

method^a^	metabolite category	specific metabolites detected
LC-MS	amino acids, lipids	glycine, serine, alanine; acylcarnitines, phosphatidylcholines^27^
	sphingolipids	C14, C16:2, C18:2 ^27^
GC-MS	phthalates	MEHP, MBP, MEP^b^ ^,^ ^29^
NMR	exploratory metabolites	1-methylnicotinamide, sphingolipids, carboxylic acids^31^
UHPLC-QE-HRMS	exposome (environmental chemicals)	pesticides, phthalates, antimicrobial agents^22^

a LC-MS: liquid chromatography–mass spectrometry; GC-MS: gas chromatography–mass spectrometry; NMR: nuclear magnetic resonance; UHPLC-QE-HRMS: ultrahigh-performance liquid chromatography Ultimate 3000 system-Q Exactive hybrid quadrupole–Orbitrap high-resolution mass spectrometry.

b MEHP: mono­(2-ethylhexyl) phthalate; MBP: mono­(*n*-butyl) phthalate; MEP: monoethyl phthalate.

### Prepregnancy BMI

Across multiple studies (Table 5), prepregnancy BMI and obesity are consistently linked to significant differences in urinary metabolite profiles during pregnancy and have been connected to health outcomes.^27–29^ One study observed lower urinary levels of glucogenic amino acids (glycine, serine, alanine) and long-chain acylcarnitines in individuals with obesity, which may reflect altered energy metabolism and a potential link to increased insulin resistance; however, these findings remain inconsistent across studies.^27^ Reduced phosphatidylcholine levels in individuals with obesity are also associated with negative impacts on fetal growth.^27^ Additionally, higher prepregnancy BMI was linked to greater perceived stress, although urinary cortisol levels were lower in pregnant women with obesity.^28^ Obesity also correlates with increased urinary phthalate metabolites such as MBP, MEP, and MEHP, as well as higher levels of inflammatory biomarkers including interleukin 1 beta (IL-1β) and C-reactive protein (CRP), highlighting the connection between BMI and inflammation.^29^ Thus, urinary metabolites differ by prepregnancy BMI though no metabolite/BMI associations overlap across studies. These findings demonstrate that prepregnancy obesity alters maternal metabolism, influencing urinary metabolite profiles and potentially impacting maternal and fetal health.

**Table 5:** Summary of Urinary Metabolites Associated with Prepregnancy BMI

BMI groups compared	key metabolites/variables	results
normal BMI vs obesity	glucogenic amino acids (glycine, serine, alanine)	lower levels of glucogenic AA in T2 and T3 in among participants with obesity, linked to insulin resistance^27^
	long-chain acylcarnitines (C14, C16:2, C18:2)	lower levels in participants with obesity, reflecting disrupted fatty acid metabolism^27^
	phosphatidylcholine species (e.g., PC aa C34:4, PC ae C38:6)	reduced levels in obesity, linked to lipid metabolism and fetal growth^27^
normal BMI vs overweight/obesity	urinary cortisol	cortisol increases blunted in participants with obesity even though women with obesity had higher perceived stress^28^
normal BMI vs obesity	phthalate metabolites (MBP, MEP, MEHP) and inflammatory biomarkers	positive associations between phthalates and inflammatory markers (IL-1β, CRP)^a^ in women with obesity^29^

a IL-1β: interleukin 1 beta; CRP: C-reactive protein.

### Pregnancy Trimester

Metabolite profiles differ across pregnancy, with the third trimester marking significant differences in molecules related to metabolism (Table 6). Urinary cortisol levels progressively increase throughout gestation, reflecting the body’s increasing metabolic and physiological demands, reaching their highest levels in the third trimester.^20^ In individuals with obesity, reductions in glucogenic amino acid concentrations were observed specifically in the second and third trimesters, suggesting that advancing pregnancy stage may contribute to these changes within this population.^27^ The third trimester shows elevated urinary excretion of metabolites linked to the pathophysiology of gestational diabetes mellitus (GDM)—such as carboxylic acids, glycerolipids, and steroid derivatives—even when managed through dietary or pharmacological interventions.^30^ Across pregnancy, trimester progression was associated with an increasing number of significantly different urinary metabolites when comparing individuals with overweight or obesity to those with normal or underweight BMI.^27^ These differences were most pronounced in the third trimester and involved metabolite classes such as acylcarnitines, biogenic amines, phosphatidylcholines and lysophosphatidylcholines, sphingomyelins, and other metabolic indicators.^27^ Gestational weight gain (GWG) was strongly associated with urinary metabolites in the second trimester, with the strongest associations involving C5 and C6 acylcarnitines and one phosphatidylcholine species (PC aa C36:5).^27^ Taurine concentrations were positively associated with GWG in both T2 and T3.^27^ Although exploratory analyses of other metabolites, such as 1-methylnicotinamide and sphingolipids, reveal some trimester-dependent fluctuations, no values showed significance.^31^ Distinct metabolite patterns have been reported in the third trimester, especially in individuals with obesity or GDM.

**Table 6:** Comparison of Urinary Metabolites Across Pregnancy Trimesters

comparison across trimesters	key metabolites/variables	results^a^
T1, T2 vs T3	cortisol (total, free, UFC)	cortisol levels progressively increase, peaking in T3 ^20^
T1, T2 vs T3	glucogenic amino acids (glycine, serine, alanine)	levels decrease significantly in T3 among obese participants, linked to insulin resistance^27^
T1, T2 vs T3	exploratory metabolites (1-methylnicotinamide, sphingolipids)	some trimester-specific variations, but no significant findings after false discovery rate correction^31^
T1, T2 vs T3	metabolite classes associated with overweight/obesity (acylcarnitines, biogenic amines, phosphatidylcholines and lysophosphatidylcholines, sphingomyelins)	more metabolites differ by BMI as pregnancy advances, with largest differences in T3 ^27^
T2 (GWG)	C5/C6 acylcarnitines, PC aa C36:5, taurine	GWG strongly associated with these metabolites in T2 ^27^
T2, T3 (GWG)	taurine	taurine positively associated with GWG in both T2 and T3 ^27^
pre- and post-GDM diagnosis	metabolites (glycerolipids, carboxylic acids, steroid derivatives)	elevated levels in T3, indicating persistent metabolic disruptions linked to pathophysiology of GDM^30^

a T1: first trimester; T2: second trimester; T3: third trimester; UFC: urinary free cortisol; GDM: gestational diabetes mellitus; GWG: gestational weight gain.

### Environmental Exposures

Exposures to dietary intake, phthalates, and other environmental factors (Table 7) are associated with distinct urinary metabolite profiles and health outcomes during pregnancy. For example, adherence to a Mediterranean diet was linked to improved sleep quality and well-being, lower stress and anxiety, and an increased cortisone/cortisol ratio.^33^ This ratio reflects greater activity of the enzyme 11β-hydroxysteroid dehydrogenase (11β-HSD), the enzyme that converts cortisol, the biologically active stress hormone, into its inactive form cortisone.^40^ An increase in the cortisone/cortisol ratio is negatively associated with perceived stress.^40^ Higher urinary glycocholate, a bile acid conjugate involved in lipid digestion and absorption, was correlated with lower α-carotene levels, emphasizing the diet’s association with urinary metabolite levels.^34^ Paraben exposure during pregnancy was associated with seven urinary metabolites, mainly diet-related, three remained significant after adjustment, with no links to endocrine disruption.^35^ Exposure to phthalates (MEHP, MBP, MEP) was associated with increased inflammatory biomarkers, but these associations were observed only in the high-BMI group.^29^ In addition, children born to women with higher prenatal urinary concentrations of phthalates, parabens, and other phenols had higher BMI z-scores and were more likely to be overweight or obese in early childhood.^36^ Urinary levels of metals such as cadmium, cobalt, copper, and vanadium were significantly associated with amino acid metabolic intermediates, including 2-oxoarginine, 3-indoleacetonitrile, and *N*-methyltryptamine, indicating alterations in tryptophan, arginine, proline, tyrosine, and lysine metabolism during pregnancy.^37^ These findings demonstrate that diet and environmental exposures are associated with urinary metabolites during pregnancy, impacting both maternal and fetal health.

**Table 7:** Urinary Metabolites Associated with Environmental and Dietary Exposures

exposure	exposed group	non-exposed group	key metabolites affected	associated outcomes
Mediterranean diet	lower stress, anxiety, improved sleep	usual care	urinary cortisone, cortisone/cortisol ratio, 5β-tetrahydrocortisone/cortisone ratio	improved mental health and well-being^33^
phthalates (MEHP, MBP, MEP)^a^	higher phthalate levels	lower phthalate levels	methyl tricosanoate, glycerolipids	increased risk of inflammation and higher BMI z-scores, increased risk of childhood overweight/obesity^29^
diet (α-carotene intake)	higher α-carotene intake	lower α-carotene intake	urinary glycocholate	negative correlation with glycocholate^34^
metals (cobalt, cadmium)	higher metal exposure	lower metal exposure	3-indoleacetonitrile, indole-5,6-quinone	not reported^37^
parabens, dichlorophenols	higher prenatal exposure	lower prenatal exposure	MEP, propylparaben, methylparaben	higher BMI z-scores, increased risk of childhood overweight/obesity^36^

a MEHP: mono­(2-ethylhexyl) phthalate; MBP: mono­(*n*-butyl) phthalate; MEP: monoethyl phthalate.

## Discussion

Herein, results from 13 published primary research articles describing urinary metabolites during pregnancy are reviewed. A synthesis of the literature in this area reveals a number of important insights. First, prepregnancy BMI is strongly linked to alterations in urinary metabolite profiles, especially in individuals with obesity.^15^ This aligns with the growing evidence that prepregnancy BMI influences metabolic pathways critical to maternal and fetal health. The third trimester marks heightened metabolic activity, including increased cortisol levels and urinary excretion of carboxylic acids, glycerolipids, and steroid derivatives.^41,42^ Next, LC-MS is the most versatile tool for detecting metabolites like amino acids and lipids, while GC-MS excels at identifying small volatile compounds like phthalates. NMR, despite lower sensitivity, provides detailed structural insights.^43,44^ Of the studies included in this review, nine utilized LC-MS and its variants (LC-MS/MS, HPLC-MS, etc.), one used GC-MS, and one applied NMR. Finally, different environmental exposures such as phthalate and metal exposures disrupt urinary metabolites and are linked to adverse health outcomes while adherence to the Mediterranean diet correlates with improved stress and higher urinary cortisone/cortisol ratio highlighting the established role of diet and environmental factors in shaping metabolic health during pregnancy.^33,45,46^ From a nutritional perspective, the results described herein demonstrate the potential of urinary metabolite profiling to guide personalized dietary and lifestyle interventions to improve the pregnancy outcomes. This review also highlights the potential of urinary biomarkers (e.g., glucogenic amino acids, long-chain acylcarnitines, and carboxylic acids) to serve as early indicators of metabolic alterations during pregnancy. While these findings may inform future prenatal care strategies, further research is necessary to establish causal relationships and their clinical utility.

Results from three primary research articles report the relationship between BMI and the urinary metabolites in nonpregnant adults.^47–49^ Higher visceral fat content was associated with urinary levels of sarcosine, trigonelline, and phenylalanine metabolites associated with insulin resistance, muscle mass and strength, and glucose metabolism and abdominal fat, respectively.^47^ BMI was also found to be associated with several urinary metabolites, such as *N*-acetyl neuraminate, trimethylamine, dimethylamine, 4-cresyl sulfate, phenylacetylglutamine, 2-hydroxyisobutyrate, succinate, citrate, ketoleucine, ethanolamine, and 3-methylhistidine. These metabolites have been associated with several key body functions such as renal function, skeletal muscle mitochondria and branched-chain amino acid metabolism, skeletal muscle turnover and meat intake, etc.^48^ This is further backed by another study in which higher BMI was associated with similar urinary metabolites, with increased levels of trimethylamine, dimethylamine, 4-cresyl sulfate, phenylacetylglutamine, 2-hydroxyisobutyrate, *N*-acetyl neuraminate, succinate, ketoleucine, 3-methylhistidine, and *N*-acetyl glycoprotein signals, and decreased levels of citrate and ethanolamine.^49^ Almost all of these metabolites with BMI were replicable across the studies, with the exception of *N*-acetyl glycoprotein signals. However, none of these specific metabolite-BMI associations observed in nonpregnant adults were reported in the studies of pregnant women included in this review. This suggests that the metabolic response to BMI may be influenced by the unique physiological state of pregnancy, supporting the need to investigate BMI-associated urinary metabolites in pregnancy as a distinct context.

Several primary research studies have compared urinary metabolites during pregnancy for individuals with and without disease for conditions other than obesity.^50–53^ It was reported that urinary phthalate metabolites such as MEP were positively associated with GDM.^50^ The prevalence of pregnancy-induced hypertension was also found to be positively associated with urinary phthalate metabolites such as MBP and MEP in multiple studies.^51,52^ Pregnancy-related hypertensive disorders, such as preeclampsia/eclampsia have been associated with increased urinary phthalate metabolites such as monobenzyl phthalate (MBzP) and mono 3-carboxypropyl phthalate (MCPP).^53^ Together, these findings suggest that elevated levels of certain urinary phthalate metabolite levels during pregnancy may serve as biomarkers of increased risk for a broad spectrum of maternal conditions, not just the variables focused on in this review.

Strengths of this study include a focus on recently published articles including most of those primary research articles published in the past 10 years (*n* = 12). Emphasizing recently published studies ensures that the findings reflect current scientific understanding and methodologies. Limiting the selection to primary research allows for direct evaluation of the original data rather than relying on secondary interpretations. These criteria enabled the inclusion of both small, detailed investigations and large-scale analyses, striking a balance between depth and generalizability. This Perspective excels in offering a holistic approach by connecting urinary metabolites to obesity status, pregnancy trimester, and environmental exposures. Limitations of this study include a language bias that may have caused the exclusion of potentially relevant research, since only studies published in English were incorporated. None of the included studies stratified obesity by BMI (I–III), despite potentially meaningful metabolic differences across obesity classes. Also, a distinct focus on specific primary exposures such as phthalates and dietary intake could have led to overlooking other exposures that may impact urinary metabolites, for instance, cannabis use. Differences in study designs, such as variation in participants’ age ranges, geographic regions, and the presence or absence of pregnancy-related health conditions (e.g., gestational diabetes), may have contributed to heterogeneity across studies. These differences could affect the comparability of urinary metabolite levels and limit the ability to synthesize consistent conclusions across populations.

Future studies should consider longitudinal analyses of urinary metabolites throughout pregnancy rather than relying on a single time point in each trimester. Such a design would enhance our understanding of the dynamic metabolic adaptations throughout pregnancy. One promising direction is to stratify analyses by prepregnancy BMI categories and link urinary metabolite patterns to child growth outcomes. This approach could help identify early metabolic signatures of pregnancy that are associated with an increased risk of childhood obesity, thereby forming early prevention strategies. Studies that integrate metabolomics with comprehensive exposome analyses could provide deeper insights into how environmental and lifestyle factors interact with maternal metabolism.^54^

This review of the literature provides evidence to support the importance of urinary metabolites as maternal metabolic health biomarkers, as demonstrated by variations in urinary metabolite profiles across prepregnancy BMI categories, pregnancy trimesters, and environmental exposures. The latest advances in metabolomics, particularly LC-MS, GC-MS, and NMR methods,^55^ provide powerful tools for detection of urinary metabolites. Such methods enable researchers to record the dynamic metabolic changes during pregnancy. In practice, these findings highlight the important role that metabolomics can play in prenatal care and public health programs focused on maternal and child health.

## References

[1] Gunderson E. P. (2009). Childbearing
and Obesity in Women: Weight Before, During, and After Pregnancy. Obstet. Gynecol. Clin. North Am..

[2] Driscoll, A. K. ; Gregory, E. C. W. Increases in Prepregnancy Obesity: United States, 2016–2019; NCHS Data Brief 392; National Center for Health Statistics, 2020. 33270551

[3] Osterman M. J. K., Hamilton B. E., Martin J. A., Driscoll A. K., Valenzuela C. P. (2025). Births:
Final Data for 2023. Natl. Vital Stat. Rep..

[4] Obesity and overweight. World Health Organization. https://www.who.int/news-room/fact-sheets/detail/obesity-and-overweight (accessed 2025-05-20).

[5] Marchi J., Berg M., Dencker A., Olander E. K., Begley C. (2015). Risks Associated
with Obesity in Pregnancy, for the Mother and Baby: A Systematic Review
of Reviews. Obes. Rev..

[6] WHO
Expert Committee on Physical Status (1995). Physical Status: The Use and Interpretation of Anthropometry.
Report of a WHO Expert Committee. W. H. O. Tech.
Rep. Ser..

[7] Bodnar L. M., Ness R. B., Markovic N., Roberts J. M. (2005). The Risk of Preeclampsia
Rises with Increasing Prepregnancy Body Mass Index. Ann. Epidemiol.

[8] Adane A. A., Tooth L. R., Mishra G. D. (2017). Pre-Pregnancy
Weight Change and Incidence
of Gestational Diabetes Mellitus: A Finding from a Prospective Cohort
Study. Diabetes Res. Clin. Pract..

[9] Souza R. T., Mayrink J., Leite D. F., Costa M. L., Calderon I. M., Rocha E. A., Vettorazzi J., Feitosa F. E., Cecatti J. G. (2019). Metabolomics
Applied to Maternal and Perinatal Health: A Review of New Frontiers
with a Translation Potential. Clinics (Sao Paulo).

[10] Guasch-Ferré M., Hruby A., Toledo E., Clish C. B., Martínez-González M. A., Salas-Salvadó J., Hu F. B. (2016). Metabolomics in Prediabetes and Diabetes:
A Systematic Review and Meta-Analysis. Diabetes
Care.

[11] Razo-Azamar M., Nambo-Venegas R., Meraz-Cruz N., Guevara-Cruz M., Ibarra-González I., Vela-Amieva M., Delgadillo-Velázquez J., Santiago X. C., Escobar R. F., Vadillo-Ortega F., Palacios-González B. (2023). An Early Prediction
Model for Gestational Diabetes Mellitus Based on Metabolomic Biomarkers. Diabetol. Metab. Syndr..

[12] Scholtens D. M., Muehlbauer M. J., Daya N. R., Stevens R. D., Dyer A. R., Lowe L. P., Metzger B. E., Newgard C. B., Bain J. R., Lowe W. L. J. (2014). Metabolomics Reveals Broad-Scale
Metabolic Perturbations
in Hyperglycemic Mothers during Pregnancy. Diabetes
Care.

[13] Hadden D. R., McLaughlin C. (2009). Normal and Abnormal Maternal Metabolism during Pregnancy. Semin. Fetal Neonatal Med..

[14] Catalano P. M. (2010). Obesity,
Insulin Resistance, and Pregnancy Outcome. Reproduction.

[15] Godfrey K. M., Reynolds R. M., Prescott S. L., Nyirenda M., Jaddoe V. W. V., Eriksson J. G., Broekman B. F. P. (2017). Influence of Maternal Obesity on
the Long-Term Health of Offspring. Lancet Diabetes
Endocrinol..

[16] Nelson S. M., Matthews P., Poston L. (2010). Maternal Metabolism
and Obesity:
Modifiable Determinants of Pregnancy Outcome. Hum. Reprod. Update.

[17] Leddy M. A., Power M. L., Schulkin J. (2008). The Impact of Maternal Obesity on
Maternal and Fetal Health. Rev. Obstet. Gynecol..

[18] Zhao H., Li H., Chung A. C. K., Xiang L., Li X., Zheng Y., Luan H., Zhu L., Liu W., Peng Y., Zhao Y., Xu S., Li Y., Cai Z. (2018). Large-Scale
Longitudinal Metabolomics Study Reveals Different Trimester-Specific
Alterations of Metabolites in Relation to Gestational Diabetes Mellitus. J. Proteome Res..

[19] Liu X., Wang X., Sun H., Guo Z., Liu X., Yuan T., Fu Y., Tang X., Li J., Sun W., Zhao W. (2019). Urinary Metabolic Variation Analysis
during Pregnancy
and Application in Gestational Diabetes Mellitus and Spontaneous Abortion
Biomarker Discovery. Sci. Rep..

[20] Jung C., Ho J. T., Torpy D. J., Rogers A., Doogue M., Lewis J. G., Czajko R. J., Inder W. J. (2011). A Longitudinal Study
of Plasma and Urinary Cortisol in Pregnancy and Postpartum. J. Clin. Endocrinol. Metab..

[21] Bouatra S., Aziat F., Mandal R., Guo A. C., Wilson M. R., Knox C., Bjorndahl T. C., Krishnamurthy R., Saleem F., Liu P., Dame Z. T., Poelzer J., Huynh J., Yallou F. S., Psychogios N., Dong E., Bogumil R., Roehring C., Wishart D. S. (2013). The Human
Urine Metabolome. PLoS One.

[22] Chen M., Guan Y., Huang R., Duan J., Zhou J., Chen T., Wang X., Xia Y., London S. J. (2022). Associations
between the Maternal Exposome and Metabolome during Pregnancy. Environ. Health Perspect..

[23] Gómez-Roig M.
D., Pascal R., Cahuana M. J., García-Algar O., Sebastiani G., Andreu-Fernández V., Martínez L., Rodríguez G., Iglesia I., Ortiz-Arrabal O., Mesa M. D., Cabero M. J., Guerra L., Llurba E., Domínguez C., Zanini M. J., Foraster M., Larqué E., Cabañas F., Lopez-Azorín M., Pérez A., Loureiro B., Pallás-Alonso C. R., Escuder-Vieco D., Vento M. (2021). Environmental Exposure during Pregnancy: Influence on Prenatal Development
and Early Life: A Comprehensive Review. Fetal
Diagn. Ther..

[24] Weldon K. C., Panitchpakdi M., Caraballo-Rodríguez A. M., Wolfe A. J., Dorrestein P. C., Brubaker L., Burnett L. A. (2025). Urinary Metabolomic
Profile Is Minimally Impacted by Common Storage Conditions and Additives. Int. Urogynecol. J..

[25] PubMed. National Center for Biotechnology Information. https://pubmed.ncbi.nlm.nih.gov.

[26] Web of Science. Clarivate. https://www.webofscience.com/.

[27] Long S. E., Jacobson M. H., Wang Y., Liu M., Afanasyeva Y., Sumner S. J., McRitchie S., Kirchner D. R., Brubaker S. G., Mehta-Lee S. S., Kahn L. G., Trasande L. (2022). Longitudinal Associations
of Pre-Pregnancy BMI and Gestational Weight Gain with Maternal Urinary
Metabolites: An NYU CHES Study. Int. J. Obes..

[28] Hohman E. E., Smyth J. M., McNitt K. M., Pauley A. M., Symons
Downs D., Savage J. S. (2023). Urinary Cortisol Is Lower in Pregnant
Women with Higher Pre-Pregnancy BMI. Front.
Endocrinol..

[29] Jin S., Cui S., Huang X., Li Z., Han Y., Cui T., Su Y., Xiong W., Zhang X. (2023). BMI-Specific Inflammatory
Response
to Phthalate Exposure in Early Pregnancy: Findings from the TMCHESC
Study. Environ. Sci. Pollut. Res. Int..

[30] López-Hernández Y., Herrera-Van Oostdam A. S., Toro-Ortiz J. C., López J. A., Salgado-Bustamante M., Murgu M., Torres-Torres L. M. (2019). Urinary
Metabolites Altered during the Third Trimester in Pregnancies Complicated
by Gestational Diabetes Mellitus: Relationship with Potential Upcoming
Metabolic Disorders. Int. J. Mol. Sci..

[31] Luthra G., Vuckovic I., Bangdiwala A., Gray H., Redmon J. B., Barrett E. S., Sathyanarayana S., Nguyen R. H. N., Swan S. H., Zhang S., Dzeja P., Macura S. I., Nair K. S. (2018). First and
Second Trimester Urinary Metabolic Profiles and Fetal Growth Restriction:
An Exploratory Nested Case-Control Study within the Infant Development
and Environment Study. BMC Pregnancy Childbirth.

[32] Irvine N., Bell R. C., Subhan F. B., Field C. J., Liu J., MacDonald A. M., Kinniburgh D. W., Martin J. W., Dewey D., England-Mason G., Kaplan B. J., Field C. J., Bell R. C., Bernier F. P., Cantell M., Casey L. M., Eliasziw M., Farmer A., Gagnon L., Giesbrecht G. F., Goonewardene L., Johnston D., Kooistra L., Letourneau N., Manca D. P., Martin J. W., McCargar L. J., O’Beirne M., Pop V. J., Deane A. J., Singhal N., Letourneau N., Bell R. C., Dewey D., Field C. J., Forbes L., Giesbrecht G., Lebel C., Leung B., McMorris C., Ross K. (2024). Maternal Pre-Pregnancy BMI Influences the Associations between Bisphenol
and Phthalate Exposures and Maternal Weight Changes and Fat Accumulation. Environ. Res..

[33] Casas I., Nakaki A., Pascal R., Castro-Barquero S., Youssef L., Genero M., Benitez L., Larroya M., Boutet M. L., Casu G., Gomez-Gomez A., Pozo O. J., Morilla I., Martínez-Àran A., Vieta E., Gómez-Roig M.
D., Casas R., Estruch R., Gratacos E., Crispi F., Crovetto F. (2023). Effects of
a Mediterranean Diet Intervention on Maternal Stress, Well-Being,
and Sleep Quality throughout Gestation-The IMPACT-BCN Trial. Nutrients.

[34] Haddad E. N., Nel N. H., Petrick L. M., Kerver J. M., Comstock S. S. (2023). Associations
between the Gut Microbiota, Urinary Metabolites, and Diet in Women
during the Third Trimester of Pregnancy. Curr.
Dev. Nutr..

[35] Rosen
Vollmar A. K., Rattray N. J. W., Cai Y., Jain A., Yan H., Deziel N. C., Calafat A. M., Wilcox A. J., Jukic A. M. Z., Johnson C. H. (2023). Urinary Paraben Concentrations and Associations with
the Periconceptional Urinary Metabolome: Untargeted and Targeted Metabolomics
Analyses of Participants from the Early Pregnancy Study. Environ. Health Perspect..

[36] Berger K., Hyland C., Ames J. L., Mora A. M., Huen K., Eskenazi B., Holland N., Harley K. G. (2021). Prenatal Exposure
to Mixtures of Phthalates, Parabens, and Other Phenols and Obesity
in Five-Year-Olds in the CHAMACOS Cohort. Int.
J. Environ. Res. Public Health.

[37] Wang M., Xia W., Liu H., Liu F., Li H., Chang H., Sun J., Liu W., Sun X., Jiang Y., Liu H., Wu C., Pan X., Li Y., Rang W., Lu S., Xu S. (2018). Urinary Metabolomics
Reveals Novel Interactions between Metal Exposure
and Amino Acid Metabolic Stress during Pregnancy. Toxicol. Res..

[38] Xia, Y. ; Sun, J. Statistical Data Analysis of Microbiomes and Metabolomics; American Chemical Society: Washington, DC, 2022. 10.1021/acsinfocus.7e5035.

[39] Nicholson J. K., Lindon J. C. (2008). Metabonomics. Nature.

[40] Shimanoe C., Matsumoto A., Hara M., Akao C., Nishida Y., Horita M., Nanri H., Higaki Y., Tanaka K. (2021). Perceived
Stress, Depressive Symptoms, and Cortisol-to-Cortisone Ratio in Spot
Urine in 6878 Older Adults. Psychoneuroendocrinology.

[41] Entringer S. (2013). Impact of
Stress and Stress Physiology during Pregnancy on Child Metabolic Function
and Obesity Risk. Curr. Opin. Clin. Nutr. Metab.
Care.

[42] Teeny S., Jarrell Z. R., Krigbaum N. Y., Cirillo P. M., Go Y.-M., Cohn B. A., Jones D. P. (2023). Third Trimester
Serum Amino Acid
Metabolism Is Associated with Maternal Breast Cancer Diagnosed within
15 Years of Pregnancy. Reseach Square.

[43] Wang X., Zhang A., Han Y., Wang P., Sun H., Song G., Dong T., Yuan Y., Yuan X., Zhang M., Xie N., Zhang H., Dong H., Dong W. (2012). Urine Metabolomics
Analysis for Biomarker Discovery and Detection
of Jaundice Syndrome in Patients with Liver Disease. Mol. Cell. Proteomics.

[44] Wang J. H., Byun J., Pennathur S. (2010). Analytical
Approaches to Metabolomics
and Applications to Systems Biology. Semin.
Nephrol..

[45] Jahan-Mihan A., Rodriguez J., Christie C., Sadeghi M., Zerbe T. (2015). The Role of
Maternal Dietary Proteins in Development of Metabolic Syndrome in
Offspring. Nutrients.

[46] Odhiambo J. F., Pankey C. L., Ghnenis A. B., Ford S. P. (2020). A Review of Maternal
Nutrition during Pregnancy and Impact on the Offspring through Development:
Evidence from Animal Models of Over- and Undernutrition. Int. J. Environ. Res. Public Health.

[47] Gurgel A. M. C., Batista A. L., Cavalcanti D. M. L. d.
P., Magalhães A., Zantut-Wittmann D. E. (2024). Sarcosine, Trigonelline and Phenylalanine as Urinary
Metabolites Related to Visceral Fat in Overweight and Obesity. Metabolites.

[48] Elliott P., Posma J. M., Chan Q., Garcia-Perez I., Wijeyesekera A., Bictash M., Ebbels T. M. D., Ueshima H., Zhao L., van Horn L., Daviglus M., Stamler J., Holmes E., Nicholson J. K. (2015). Urinary Metabolic Signatures of Human
Adiposity. Sci. Transl. Med..

[49] Wang T., Tang L., Lin R., He D., Wu Y., Zhang Y., Yang P., He J. (2021). Individual
Variability
in Human Urinary Metabolites Identifies Age-Related, Body Mass Index-Related,
and Sex-Related Biomarkers. Mol. Genet. Genomic
Med..

[50] Shaffer R. M., Ferguson K. K., Sheppard L., James-Todd T., Butts S., Chandrasekaran S., Swan S. H., Barrett E. S., Nguyen R., Bush N., McElrath T. F., Sathyanarayana S. (2019). Maternal Urinary
Phthalate Metabolites in Relation to Gestational Diabetes and Glucose
Intolerance during Pregnancy. Environ. Int..

[51] Bedell S. M., Lyden G. R., Sathyanarayana S., Barrett E. S., Ferguson K. K., Santilli A., Bush N. R., Swan S. H., McElrath T. F., Nguyen R. H. N. (2021). First- and Third-Trimester
Urinary Phthalate Metabolites
in the Development of Hypertensive Diseases of Pregnancy. Int. J. Environ. Res. Public Health.

[52] Soomro M. H., Maesano C. N., Heude B., Bornehag C.-G., Annesi-Maesano I. (2021). The Association
between Maternal Urinary Phthalate Metabolites Concentrations and
Pregnancy Induced Hypertension: Results from the EDEN Mother-Child
Cohort. J. Gynecol. Obstet. Hum. Reprod..

[53] Meeker J. D., McArthur K. L., Adibi J. J., Alshawabkeh A. N., Barrett E. S., Brubaker S. G., Cordero J. F., Dabelea D., Dunlop A. L., Herbstman J. B., Kahn L. G., Karr C. J., Mehta-Lee S., O’Connor T. G., Sathyanarayana S., Trasande L., Kuiper J. R. (2024). Urinary Concentrations of Phthalate
Metabolites in Relation to Preeclampsia and Other Hypertensive Disorders
of Pregnancy in the Environmental Influences on Child Health Outcomes
(ECHO) Program. Environ. Int..

[54] Vrijheid M. (2014). The Exposome:
A New Paradigm to Study the Impact of Environment on Health. Thorax.

[55] Liu R., Bao Z.-X., Zhao P.-J., Li G.-H. (2021). Advances in the
Study of Metabolomics and Metabolites in Some Species Interactions. Molecules.

